# Tobacco control policies to promote awareness and smoke-free environments in residence and workplace to reduce passive tobacco smoking in Bangladesh and its correlates

**DOI:** 10.1371/journal.pone.0198942

**Published:** 2018-06-19

**Authors:** Papia Sultana, Md. Tahidur Rahman, Dulal Chandra Roy, Shamima Akter, Jenny Jung, Md. Mizanur Rahman, Jahanara Akter

**Affiliations:** 1 Department of Statistics, University of Rajshahi, Rajshahi, Bangladesh; 2 Safi Consultancy, Sylhet, Bangladesh; 3 Department of Epidemiology and Prevention, Clinical Research Center, National Center for Global Health and Medicine, Tokyo, Japan; 4 Graduate School of Arts and Sciences, Yale University, New Haven, CT, United States of America; 5 Global Public Health Research Foundations, Dhaka, Bangladesh; 6 Department of Global Health Policy, The University of Tokyo, Tokyo, Japan; 7 Department of Population Science and Human Resource Development, University of Rajshahi, Rajshahi, Bangladesh; Johns Hopkins University, UNITED STATES

## Abstract

**Background:**

Bangladesh is one of the highest tobacco consuming countries in the world, with reported 21.2% of the population as daily smokers, 24.3% as smokeless tobacco users, and 36.3% as adult passive smoker. Given the high prevalence and established harmful effects of passive tobacco smoking, this study aimed to estimate of pattern of smoking policies in residential and work place, and to identify the associated socio-economic and demographic correlates in Bangladesh.

**Data and methods:**

Secondary data of sample size 9629 collected by the Global Adult Tobacco Survey (GATS) 2010 has been used. Along with descriptive analysis, binary logistic regression model has been used to analyze the socio-demographic and economic correlates to tobacco smoking policy.

**Results:**

The prevalence of male and female passive tobacco smokers was 74.3% and 25.8% respectively. Among the passive tobacco smokers, 22.2% reported that smoking was allowed at their home and 29.8% reported that there was no such smoking policy at their home. Alternatively, 26.0% passive tobacco smokers reported that smoking was allowed and 27.5% reported that there was no such smoking policy at their work place. Logistic regression analysis indicated that for tobacco smokers group, the odds of allowing smoking at home was 4.85 times higher than the non-smoker respondent (OR = 4.85, 95% CI = 4.13, 5.71), 1.18 times more likely to be allowed at home in rural areas than urban areas (OR = 1.18, 95% CI = 1.06,1.32) and less for college/university completed and (or) higher educated respondent than no formal schooling (OR = 0.35, 95% CI = 0.24, 0.52). On the other hand, smoking was 1.70 times more likely to be allowed at work place for tobacco smokers than their counter part respondent (OR = 1.70, 95% CI = 1.36, 2.14) and was less likely to be allowed for college/university completed and (or) higher educated respondent (OR = 0.26, 95% CI = 0.14, 0.45) than respondent with no formal schooling.

**Conclusion:**

To reduce the passive smoking, lower educated people and people in urban areas should advocate more about the adverse effect of active and passive tobacco smoking. Also, smoking policy should reform introducing smoking zone at work places and residential buildings.

## Introduction

Bangladesh is one of the top countries with high smoking prevalence countries in the world. Approximately 48.3% of men and 1.5% of women have reported to smoke some form of tobacco product on a daily or occasional basis in Bangladesh [1]. Smoking attributed to 25% of all deaths in Bangladeshi men aged 25 to 69 years and resulted in an average of seven years of life lost per smoker [2]. A recent study by Rahman and colleagues reported a total prevalence of 21.2% who are daily smokers, 24.3% consume smokeless tobacco products, and 36.3% are adult passive smokers [3]. This implies that for every 100 direct tobacco smokers create 172 passive tobacco smokers. Passive tobacco smoking, defined as the exposure to second-hand tobacco smoke, is linked to several harmful health outcomes such as respiratory infections, ischemic heart disease, lung cancer, and asthma. Passive tobacco smoking is as harmful as direct tobacco smoking [4–9] and affects predominantly children or women. Tobacco smoking is least prevalent in women due to social norms; however 14.3% of women are exposed to passive tobacco smoking [3]. Another population group who bear high burden of second hand smoking is children, and a recent survey found 95% of primary school children in Dhaka had recently been exposed to second hand smoke. Despite government efforts to protect individuals from exposure to tobacco smoke, such as ratification of World Health Organization Framework Convention on Tobacco Control (WHO FCTC) and The Bangladesh Tobacco Control Act 2005, smoking on indoor public/workplaces and public transportation remain a common behavior. This reflects the importance to research the extent of exposure to second hand smoking and the related sociodemographic and economic factors to promote effective policy interventions in Bangladesh.

To our knowledge, several studies have been conducted on tobacco smoking in Bangladesh [1–3, 10–20]. However, most of these previous studies have been limited to the prevalence and predictors of tobacco use [1, 13–19]. Few studies address the economic issue of tobacco use [10, 20] and issue of knowledge and attitude [3]. Therefore, this study will be the first attempt to consider the effects of smoking policy in home and at work place in Bangladesh. The aim of our study was to obtain a nationally representative estimate of pattern of smoking policy at home and work place in Bangladesh, and to identify socio-economic and demographic correlates.

## Data

We extracted secondary data collected by the Global Adult Tobacco Survey (GATS), 2010 [21]. The survey was conducted in 14 countries including Bangladesh, Brazil, China, Egypt, India, Mexico, Philippines, Poland, Russia, Thailand, Turkey, Ukraine, Uruguay and Vietnam from 2008 to 2010. GATS used a global standardized methodology. Details about the survey design, survey methods, questionnaire, and definitions of various terminologies can be found in [21–24]. The wealth index was constructed by the GATS Collaborator Team using principal component analysis (PCA) method [2, 24].

## Statistical methods

Various statistical methodologies have been used to analyze the data. Descriptive analysis has been performed to know the characteristics of the study subjects. For that frequency with percentage or mean with standard deviation has been reported, whichever applicable. A comparison of socio-demographic and economic characteristics of study subjects to confounding variables (residence and gender) and to the outcome variable (smoking policy) have been done. To compare variables chi-square test (Pearson Chi-square or Likelihood Ratio Chi-square) has been used for categorical data, and prevalence with 95% confidence interval has been reported for individual variable. On the other hand, t-test to compare mean has been used for continuous data and mean with standard deviation has been reported [25]. These tests have been performed at 5% level of significance. To analyze the socio-demographic and economic correlates to tobacco smoking policy, binary logistic regression has been used and Odds Ratio (OR) with 95% confidence interval has been reported [26]. Statistical software StataSE version 11 (StataCorp, USA) has been used to carry out statistical analyses. Missing data of “age” and “occupation” has been adjusted using related information [2,3].

## Results

The total sample size of 9629 of which 4468 (46.4%) were male and 5161 (53.6%) were female. Approximately 47% (n = 4550) of total respondents reported themselves as a passive smoker, which was higher in males (n = 3381, 74.3%) compared to females (n = 1169, 25.8%) (Table 1). Although not shown in the table, we found that among the female passive smokers, 21.4% were in homes and 18.9% were from the workplace.

**Table 1:** Characteristics of the study subjects.

Characteristics	Total respondent (n = 9629) (n, %)	Passive smoker (n = 4550) (n, %)
** Gender**		
Male	4468 (46.40)	3381 (74.31)
Female	5161 (53.60)	1169 (25.79)
** Place of Residence**		
Urban	4857 (50.44)	2401(52.77)
Rural	4772 (49.56)	2149(47.23)
** Mean age (years, SD)**	36.90 (14.90)	36.30 (13.65)
** Educational level**		
No formal schooling	3430 (35.62)	1425 (31.32)
Less than primary school completed	1487 (15.44)	711 (15.63)
Primary school completed	1115 (11.58)	502 (11.03)
Less than secondary school completed	1937 (20.12)	943 (20.73)
Secondary school completed	663 (6.89)	354 (7.78)
High school completed	463 (4.81)	271 (5.96)
Tertiary education completed or higher	484 (5.03)	338 (7.43)
Don’t know	50 (0.52)	6 (0.13)
** Occupational category**		
Employment (Govt, NGO)	961 (9.98)	674 (14.81)
Business (Small or large)	993 (10.31)	851 (18.70)
Farming (land owner & farmer)	826 (8.58)	582 (12.79)
Agricultural / Industrial worker/ daily laborer/other self- employed	1537 (15.96)	998 (21.93)
Homemaker/Housework	4030 (41.85)	833 (18.31)
Retired and unemployed (able to work/unable to work)	431 (4.48)	145 (3.19)
Student/Other	851 (8.84)	467 (10.26)
** Wealth index**		
Q_1_ (Poorest)	1866 (19.38)	720 (15.82)
Q_2_	2068 (21.48)	917 (20.15)
Q_3_	1732 (17.99)	821 (18.04)
Q_4_	2040 (21.19)	1064 (23.38)
Q_5_ (Richest)	1923 (19.97)	1028 (22.59)

Wealth index was calculated by PCA method using household items, number of rooms used for sleeping and materials of roof of the respondents.

**Fig 1:**
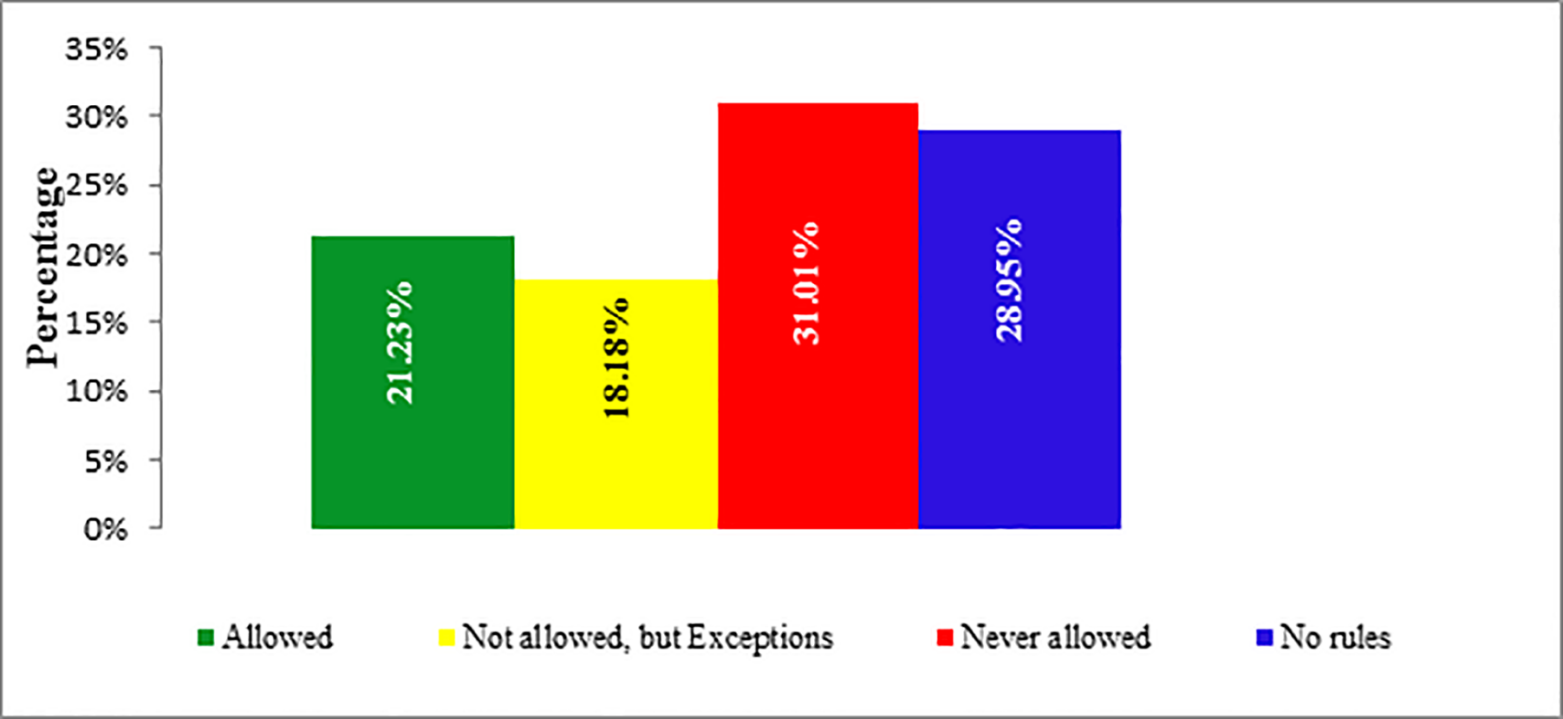
Smoking policy at home.

Table 2 reports policies as stated by passive smokers in homes and workplace. In the home, the most common policy was that smoking was never allowed (n = 1409, 30.97%), followed by no rules (n = 1347, 29.82%), and smoking was allowed (n = 22.15%). Valid prevalence of smoking policies at home is also presented in bar diagram (Fig 1).

**Table 2:** Smoking policy at home and workplace as reported by passive smokers.

	Passive smoker (n = 4550)
**Smoking policy at home (%)**	
Allowed	1008(22.15)
Not allowed, but exceptions	739 (16.24)
Never allowed	1409(30.97)
No rules	1357(29.82)
Don’t know	36 (0.79)
Refused	1 (0.02)
**Smoking policy at the work place (%)**	
Allowed anywhere	479 (26.03)
Allowed only in some indoor areas	273 (14.84)
Not allowed at all	545 (29.62)
No rules	506 (27.50)
Don’t know	36 (1.96)
Refused	1 (0.05)

**Fig 2:**
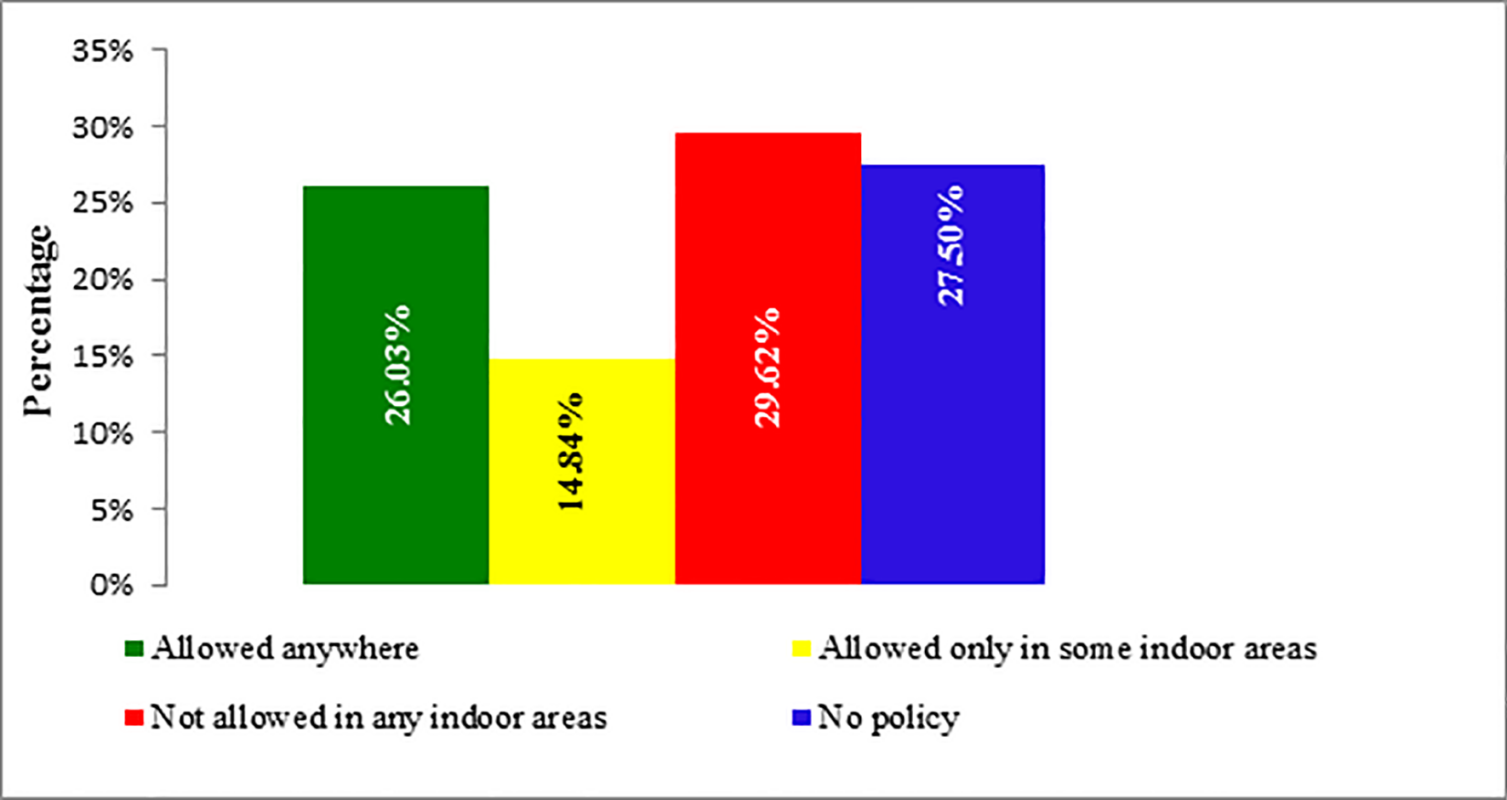
Smoking policy at work place.

Similarly, in the workplace the most common policy was to ban smoking (n = 545, 29.62%), followed by no rules in place (n = 506, 27.50%), and smoking was allowed (n = 479, 26.03%). On the other hand, 26.0% passive tobacco smokers reported that smoking was allowed at their job place and 27.5% reported that there was no such smoking policy at their job place (Table 2). Valid prevalence of smoking policies at work place is also presented in bar diagram (Fig 2).

Among passive smokers, smoking policy at home and at work place significantly differ by gender and by residence (Tables 3 and 4). It has been also found that a significant percentage was female among the respondents who become passive smoker due to smoking was allowed in home (19.3%) and at work place (5.9%) (Table 5).

**Table 3:** Smoking policies according to gender among passive smokers.

Characteristics	Male	Female	p-value*
**Smoking policy at home (%, 95% CI)**			
Allowed	20.24 (18.87–21.61)	22.82 (19.71–25.93)	<0.001
Not allowed, but exceptions	18.30 (16.98–19.62)	14.40 (11.80–17.01)	
Never allowed	32.91 (31.31–34.52)	30.52 (27.11–33.94)	
No rules	28.41 (26.87–29.94)	25.52 (26.14–32.90)	
**Smoking policy at work place (%)**			
Allowed anywhere	26.48 (24.38–28.59)	16.56 (10.94–22.19)	0.014
Allowed only in some indoor areas	15.04 (13.34–16.74)	10.65 (5.98–15.31)	
Not allowed at all	30.08 (27.90–32.27)	37.27 (29.96–44.59)	
No rules	27.78 (24.67–28.89)	31.36 (24.34–38.38)	

*p-value has been calculated from chi-square test for association.

Note: Don’t know and refused have been considered as missing.

**Table 4:** Smoking policies by residence among passive smokers.

Characteristics	Urban	Rural	p-value*
**Smoking policy at home (%, 95% CI)**			
Allowed	18.52 (16.90–20.13)	23.42 (21.45–25.39)	<0.001
Not allowed, but exceptions	18.20 (16.60–19.80)	16.89 (15.14–18.63)	
Never allowed	36.99 (34.99–39.00)	26.85 (24.79–28.92)	
No rules	25.96 (24.14–27.78)	31.92 (29.75–34.09)	
**Smoking policy at work place (%)**			
Allowed anywhere	24.93 (22.50–27.37)	26.81 (23.39–30.22)	0.001
Allowed only in some indoor Areas	15.39 (13.35–17.42)	13.25 (10.63–15.86)	
Not allowed at all	33.41 (30.76–36.07)	25.73 (22.36–29.09)	
No rules	24.03 (21.62–26.43)	33.12 (29.50–36.75)	

*p-value has been calculated from chi-square test for association.

Note: Don’t know and refused have been considered as missing.

**Table 5:** Comparing various cofactors to smoking policy as reported by passive smokers.

Socioeconomic and demographic variables	Smoking policy at home (n, 95% CI)				Smoking policy at work place(n, 95% CI)			
	Allowed (N = 743)	Not allowed, but exceptions (N = 636)	Never allowed (N = 1085)	No rules (N = 1013)	Allowed anywhere (N = 475)	Allowed only in some indoor areas (N = 268)	Not allowed at all (N = 473)	No rules (N = 481)
**Residence**								
Urban	49.82 (46.41,53.22)	57.51 (52.67,61.16)	63.36 (60.74, 65.98)	50.52 (47.63,53.42)	63.52 (59.19,67.84)	68.49 (62.97,74.022)	70.85 (67.12,74.58)	57.59 (53.28,61.90)
Rural	50.18 (46.77,53.58)	42.49 (38.84,47.33)	36.64 (34.01,39.25)	49.48 (46.57,52.37)	36.48 (32.15,40.80)	31.51 (25.97,37.02)	29.15 (25.41,32.87)	42.41 (38.09,46.71)
**Gender**								
Male	80.70 (78.01,83.38)	85.69 (83.10, 88.27)	83.56 (81.54,85.57)	81.93 (79.70,84.16)	94.12 (92.01,96.24)	93.41 (90.45,96.35)	89.01 (86.43,91.57)	89.55 (86.87,92.21)
Female	19.30 (16.61,21.98)	14.31 (11.72,16.89)	16.44 (14.42,18.45)	18.07 (15.83,20.29)	5.88 (3.75,7.98)	6.59 (3.64,9.54)	10.99 (8.42,13.56)	10.45 (7.78,13.12)
**Mean age (years, SD)**	37.90 (0.49)	36.08 (0.51)	36.08 (0.41)	36.49 (0.43)	36.15 (0.56)	37.70 (0.76)	35.56 (0.55)	38.08 (0.61)
**Educational level**								
No formal schooling	44.75 (41.36,48.14)	25.49 (22.27,28.71)	18.04 (15.95,20.13)	32.95 (30.22,35.68)	31.44 (27.27,35.62)	19.41 (14.71,24.11)	12.56 (9.84,1528)	26.48 (22.63,30.33)
Less than primary school completed	16.76 (14.22,19.31)	16.57 (13.82, 9.31)	13.51 (11.65,15.37)	17.65 (15.44,19.86)	17.81 (14.37,21.25)	13.18 (9.16,17.21)	12.21 (9.53,14.90)	16.79 (13.53,20.06)
Primary school completed	8.68 (6.76, 10.60)	9.63 (7.45,11.81)	10.59 (8.92, 12.27)	12.32 (10.4, 14.23)	12.36 (9.40,15.32)	10.25 (6.64,13.86)	9.07 (6.71,11.43)	11.06 (8.32,13.80)
Less than secondary school completed	17.61 (15.01,20.20)	21.67 (18.62,24.71)	21.88 (19.64,24.13)	20.45 (18.11,22.79)	20.75 (17.10,24.40)	15.38 (11.09,19.67)	21.29 (17.93,24.64)	23.71 (20.00,27.42)
Secondary school completed	5.18 (3.67, 6.69)	8.64 (5.56,10.71)	11.36 (9.64, 13.09)	7.08 (5.59, 8.56)	7.96 (5.53,10.40)	12.08 (8.21,15.96)	11.34 (8.74,13.94)	7.70 (5.37,10.03)
High school completed	3.86 (2.54, 5.17)	8.07 (6.06, 10.08)	9.37 (7.78, 10.95)	4.37 (3.18, 5.55)	5.03 (3.06,6.99)	8.05 (4.82,11.29)	9.94 (7.49,12.40)	6.32 (4.19,8.44)
Tertiary education completed or higher	3.13 (1.94, 4.32)	9.91 (7.70, 12.12)	15.20 (13.25,17.15)	5.15 (3.87, 6.43)	4.61 (2.72,6.49)	21.61 (16.71,26.50)	23.56 (20.08, 27.04)	7.90 (5.55,10.25)
**Occupational Category**								
Employment (Gov, NGO)	11.94 (9.73, 14.15)	17.42 (14.62,20.22)	24.73 (22.38,27.07)	12.91 (10.97,14.85)	15.93 (12.64,19.22)	39.56 (33.74,45.37)	55.84 (51.77,59.91)	14.99 (11.87,18.10)
Business (small, large)	20.50 (17.75,23.25)	22.52 (19.43,25.60)	21.88 (19.64,24.13)	20.41 (18.08,22.75)	39.41 (35.02,43.80)	22.34 (17.39,27.29)	17.97 (14.82,21.12)	38.46 (34.21,42.70)
Farming (land owner & farmer)	14.95 (12.52,17.38)	14.73 (12.11,17.34)	10.90 (9.21, 12.60)	13.43 (11.46,15.41)	7.96 (5.53,10.40)	8.42 (5.12,11.72)	6.80 (4.74,8.87)	7.10 (4.86,9.33)
Agricultural / Industrial worker/ daily laborer/Other self- employed	28.70 (25.62,31.79)	24.36 (21.19,27.53)	15.89 (13.91,17.88)	27.57 (24.98,30.16)	29.97 (25.86,34.09)	25.27 (20.10,30.44)	10.12 (7.64,12.69)	27.81 (23.90,31.71)
Homemaker/Housework	12.30 (10.06,14.54)	8.07 (6.06, 10.08)	10.21 (8.56, 11.86)	11.95 (10.07,13.83)	1.04 (0.13,1.96)	0.36 (0.35,1.08)	0.69 (0.01,1.38)	1.97 (0.76,3.18)
Retired and unemployed (able to work/unable to work)	3.13 (1.94, 4.32)	2.69 (1.49,3.88)	3.99 (2.92,5.05)	3.05 (2.05, 4.05)	—	0.36 (0.35,1.08)	0.52 (0.06,1.11)	1.57 (0.49,2.66)
Student/Other	8.44 (6.54,10.33)	10.19 (7.96,12.43)	12.36 (10.57,14.15)	10.64 (8.85,12.43)	5.66 (3.58,7.73)	3.66 (1.42,5.89)	8.02 (5.79,10.25)	8.08 (5.70,10.46)
**Wealth index**								
Q_1_ (Poorest)	20.98 (18.21,23.76)	12.03 (9.63, 14.44)	7.75 (6.30, 9.21)	20.06 (17.74,22.39)	13.20 (10.16,16.25)	10.25 (6.64,13.86)	3.66 (2.12,5.20)	13.01 (10.08,15.95)
Q_2_	24.12 (21.21,27.04)	17.70 (14.88,22.52)	14.66 (12.74,16.59)	21.29 (28.91,23.66)	17.61 (14.18,21.03)	19.04 (14.37,23.71)	13.08 (10.32,15.85)	17.75 (14.41,2108)
Q_3_	18.09 (15.47, 20.71)	17.70 (14.88,20.52)	16.51 (14.49, 8.53)	18.41 (16.16,20.65)	20.54 (16.91,24.17)	11.35 (7.58,15.12)	15.88 (12.88,18.87)	17.15 (13.87,20.44)
Q_4_	37.76 (20.86,26.66)	24.36 (21.19,27.53)	25.42 (23.05,27.78)	22.16 (19.75,24.57)	28.30 (24.25,32.35)	20.51 (15.71,25.31)	27.22 (23.57,30.87)	26.23 (22.39,30.06)
Q_5_ (Richest)	13.02 (10.73, 15.32)	28.18 (24.86,31.50)	35.63 (33.03,38.24)	18.06 (15.83,20.29)	20.33 (16.71,23.95)	38.82 (33.03,44.62)	40.13 (36.11,44.15)	25.83 (22.02,29.65)

— Data not available; Note: Don’t know and refused have been considered as missing.

Risk factors assessment for tobacco smoking policies is presented in Table 6. Logistic regression analysis indicated that for tobacco smokers smoking was 4.85 times more likely to be allowed at home than non-smoker respondent (OR = 4.85, 95% CI = 4.13–5.71). Smoking was 1.18 times more likely to be allowed at home in rural areas than urban areas (OR = 1.18, 95% CI = 1.06–1.32). Again smoking was less likely to be allowed at home for respondent with college/university completed or higher than respondent with no formal schooling (OR = 0.35, 95% CI = 0.24, 0.52), inversely smoking was 2.85 times more likely to be allowed at home for respondent with no formal schooling than respondent with college/university completed or higher. Wealthy population was less likely to allow smoking at home than disadvantaged population (OR = 0.60, 95% CI = 0.49–0.74).

**Table 6:** Identifying correlates of smoking policy at home and work place using binary logistic regression.

Socio- demographic and economic variables	Smoking policy OR (95% CI)	
	Smoking allowed at home	Smoking allowed at work place
**Tobacco Smoker**	4.85(4.13,5.71)*	1.70(1.36,2.14)*
**Residence**		
Urban(RC)	1.00	1.00
Rural	1.18(1.06,1.32)*	0.80(0.63, 1.01)
**Gender**		
Male(RC)	1.00	1.00
Female	2.74(2.26,3.32)*	0.34(0.22,0.53)*
**Respondent Age (yrs)**	0.99(0.99,1.00)	0.98(0.97, 1.00)
**Educational level**		
No formal schooling(RC)	1.00	1.00
Less than primary school completed	0.78(0.67,0.90)*	0.70(0.50,0.98)*
Primary school completed	0.71(0.60,0.82)*	0.73(0.50, 0.99)*
Less than secondary school completed	0.64(0.55,0.75)*	0.58(0.41,0.83)*
Secondary school completed	0.56(0.44,0.72)*	0.56(0.35,0.89)*
High school completed	0.58(0.43,0.80)*	0.45(0.26,0.78)*
Tertiary education completed or higher	0.35(0.24,0.52)*	0.26(0.14,0.45)*
**Occupational category**		
Employment (Gov, NGO) (RC)	1.00	1.00
Business (small, large)	1.12(0.86,1.147)	2.96(2.17,4.03)*
Farming (land owner & farmer)	1.01(0.78,1.37)	1.97(1.20,3.20)*
Agricultural / Industrial worker/ daily laborer/Other self- employed	1.09(0.85,1.39)	2.33(1.67,3.28)*
Homemaker/Housework	1.36(1.06,1.74)	2.25(0.79,6.39)
Retired and unemployed (able to work/unable to work)	1.03(0.73,1.45)	—
Student/Other	1.57(1.20,2.05)*	1.47(0.90,2.41)
**Wealth index**		
Q_1_ (Poorest)	1.00	1.00
Q_2_	0.94(0.82, 1.09)	0.79(0.53,1.17)
Q_3_	0.92(0.78,1.07)	1.05(0.70,1.57)
Q_4_	0.90(0.89,1.07)	1.03(0.69, 1.55)
Q_5_ (Richest)	0.60(0.49,0.74)*	0.84(0.52,1.33)
P-value from Hosmer-Lemeshow goodness of fit test:	0.7216	0.6893
Prediction accuracy (AUC):	0.6648	0.6141

RC: Reference category,—Data not available, AUC: Area Under the ROC curve

* statistically significant

Note: Don’t know and refused have been considered as missing.

Smoking was 1.70 times more likely to be allowed at work place for tobacco smokers than their counter part respondent (OR = 1.70, 95% CI = 1.36–2.14). Smoking was less likely to be allowed at work place for respondent with college/university completed and (or) higher than respondent with no formal schooling (OR = 0.26, 95% CI = 0.14–0.45), inversely smoking was about 4 times more likely to be allowed at work place for respondent with no formal schooling than respondent with college/university completed and (or) higher.

## Discussion

In this study it has been found that a significant proportion of passive smoking occurs due to smoking was allowed at home and work place. In developed countries, there are some smoking zones at offices and no smoking is allowed at home. In developing countries like Bangladesh, there is no evidence about smoking zone at office. Although this study has found that government and non-government working places are less likely to allow smoking than other occupational working places. This might be due to health awareness of those government and non-government employees. Usually higher educated peoples are involved in employment and they are less likely to allow smoking at home and work place than lower educated people. This is obvious and expectable. Like other neighboring countries [27, 28], higher education plays an important role to have lower degree of fatalism and overall risk taking behavior in Bangladesh, too. Educated peoples are more aware about health, as well as more aware about social and official norms. On the other hand, wealthy respondent was less likely to allow smoking at home than disadvantaged respondent; however, no specific pattern was found to allow smoking at work place regarding the wealth index.

## Strength and limitation

The major strengths of our study are nationally representative population-based survey and the coverage of both male and female including urban and rural areas. To our knowledge none of the earlier studies had captured comprehensive information on tobacco smoking policy in home and at work place in Bangladesh. Therefore, the present study may have great bearing on the public health policy. Some common limitations of the survey have been discussed in [2]. However, in constructing wealth index, a single asset index was developed for the whole sample; indices were not prepared for urban and rural populations separately [18, 21]. The study was of cross-sectional nature. Therefore, we could not assess the trend of smoking policy at home and work place in Bangladesh.

## Conclusion

This study clearly revealed that passive smoking is highly associated with smoking policy at home and work place. Therefore, smoking policy should reform introducing smoking zone at work places and residential buildings. Government may enforce to establish specific smoking venue in residential apartment and in hospitality areas in workplaces [29–32]. In developed countries smoking venues are modern and safe with area>100m^2^, filtered and with air ventilation. In addition, to reduce the passive smoking, lower educated people and people in urban areas should advocate more about the adverse effect of active and passive smoking. In addition, they should be light up from inside about the social manner, especially not to smoke in front of nonsmokers and children. More advertisement, community programs, etc. on adverse effect of active and passive tobacco smoking would be effective to advocate lower educated people. Besides electronic and print media, advertisement might be on billboard, wall, back side of *rickshaw*, *auto*, and other vehicles. Community programs may include theater show, arrangement of workshops, speech on it few minutes before *Khutba* on Friday’s prayer by community leaders, etc. Other existed tobacco control policies, like banning smoking in public places should be strengthen more.
